#  Assessment of Anthropometric Indices in Patients with Phenylketonuria

**Published:** 2020

**Authors:** Marjan SHAKIBA, Mohammadreza Alaei, Hedyeh SANEIFARD, Asieh MOSALLANEJAD

**Affiliations:** 1 Department of Pediatric Endocrinology and Metabolic diseases, Mofid children hospital, Shahid Beheshti University of Medical Sciences, Tehran, Iran.

## Abstract

**Objectives:**

Dietary phenylalanine restriction is the main treatment of phenylketonuria (PKU, OMIM 261600). There are a number of studies which have demonstrated growth retardation in these patients, and some are in contrast. This study was performed to assess the growth parameters of treated PKU patients.

**Materials & Methods:**

This cross-sectional study was performed between 2015 and 2017 to compare growth indices in PKU patients in our clinics with normal age and sex matched controls. Weight, height, head circumference (HC), weight for height and BMI (weight/height^2^) were measured and converted into Z-scores. We assessed differences between patients and controls’ anthropometric indexes in all patients and separately in patients who were diagnosed by newborn screening program and patients who were diagnosed after presentation of clinical manifestations in comparison with age and sex-matched controls. Also, this difference was assessed separately in patients aged two years and less. Correlations between pretreatment plasma phenylalanine concentrations mean plasma phenylalanine concentrations and anthropometric parameters were analyzed in the patients.

**Results:**

Overall*, *209 under-treatment PKU patients (103 males, 106 females; mean age 9.29 ± 8.7 years) and 216 controls (109 males and 107 females; mean age 8.98 ± 8.62 years) matched in terms of age, sex and birth weight were enrolled in this study. In general, 130 patients were diagnosed by newborn screening and 79 were diagnosed when they became symptomatic before the screening program. A significant difference (p=0.000) was found only in HC z-score and weight for height z-score in comparison with the control group, when we assessed all patients. We did not find any significant differences in any of the anthropometric indexes between cases and controls who were aged 2 years old and less. Head circumference SDS and weight for height SDS were significantly different when patients and controls who were more than 2 years old were compared. Mean HC was significantly lower in patients, while BMI SDS, weight SDS, and weight for height SDS were significantly higher in PKU patients in comparison with the control group when patients who were diagnosed in newborn screening were assessed. Head circumference SDS, BMI, height SDS and difference between patients’ height SDS and mid parental height SDS had significantly lower mean scores in comparison with those of the control group, while mean weight SDS was significantly higher compared to controls when patients who were diagnosed after clinical presentation were assessed.

Mean phenylalanine was not correlated with anthropometric indices, while there was a correlation between pretreatment phenylalanine and HC.

**Conclusion:**

Disparities in anthropometric indexes changes observed in different studies may be due to diverse diet protocols, availability of various specific products and micronutrient substitutes.

## Introduction

Phenylketonuria (PKU) stems from phenylalanine hydroxylase enzyme deficiency where the essential amino acid phenylalanine (Phe) is not converted into tyrosine. Accumulation of phenylalanine and its metabolites damages developing neural system in patients. 

Despite new approaches for the treatment of PKU, natural protein and phenylalanine-restricted diet and administration of Phe free protein substitutes for PKU patients are the main treatments for achieving normal plasma phenylalanine and preventing complications of high phenylalanine levels, such as mental retardation, although some degree of neuropsychological dysfunction may remain (1, 2). Also, adhering to this kind of diet results in a number of complications such as micronutrients, minerals, vitamins and essential fatty acids deficiency; thus, each patient's dietary habits should be considered to acquire milestones and have a normal growth (3-5).

Several studies have been designed for the assessment of growth in PKU patients and finding the effects of Phe-restricted diet on physical growth of PKU patients under treatment, but inconsistent findings have been achieved (6-10). 

Herein, we measured growth parameters, weight for height and BMI in patients who were under Phe-restricted diet. We also considered familial and constitutional short stature in these patients. Furthermore, we examined the correlation between patients' physical growth and their pretreatment and mean plasma phenylalanine concentrations, which are signs of disease severity and dietary adherence, respectively.

## Patients and Methods:

This cross-sectional study was performed from 2015 to 2017 in PKU clinics in Mofid Children’s Hospital, where the patients were followed up (a referral hospital). We considered a patient to have PKU if plasma phenylalanine concentrations were > 6 mg/dl in untreated newborn infants (child). The study was approved by the Ethics Committee of Shahid Beheshti University of Medical Sciences.

Z-score of head circumference (HC), body mass index (BMI: weight/height^2^), weigh, and height were compared between PKU patients matched in terms of age, sex and birth weight. We selected age and sex-matched controls from normal children who visited our endocrine clinics for following their growth and they are completely normal without any underlying or chronic diseases. For participants with age more than 20 years, we selected controls from our medical staff and medical students.

We performed height measurement for patients who were more than 2 years old with wall-mounted stadiometer, asking the patient to stand erect, remove bulky clothing, hair ornaments and shoes, put heels together and arms at sides, look straight ahead so that the line of sight was parallel with the floor. Recumbent length measurements for children less than 2 years old were done by an infantometer. Weight measurements were performed by Seca mechanical medical and baby scales. Occipitofrontal circumference, (circumference of the head around the occiput of the skull to the most anterior portion of the frontal bone), were measured as HC, by a flexible tape measure.

All the measurements were performed by a single clinical physician and same measure instruments.

Weight, height, HC (< 3 years), weight for height (<2 y/o) and BMI (>2 y/o) were tracked into NCHS centiles. Head circumference for the patients above 3 y/o were tracked to charts, modified from Nelhaus G, J pediatr 1968;48:106 (11).

As the measurements were not performed at the same ages in patients, height, weight and HC were transformed into Z-scores by subtracting the patient's measurements from expected mean measurement for those of age and sex, and dividing it by its standard deviation. Z-scores between -2 and +2 considered as normal range. 

Furthermore, correlation between pretreatment plasma Phe concentrations, mean plasma Phe concentrations during treatment, and anthropometric parameters were analyzed in the patients.

Initial dietary protocols depended on the maximum pretreatment Phe plasma levels (70 mg/kg/day phenylalanine was recommended for the initial plasma Phe <10 mg/dl ,55 mg/kg/day for plasma Phe 10-20, 45 mg/kg/day for 20-30 mg/dl, 35 mg/kg/day for 30-40 mg/dl and 25 mg/kg/day for initial Phe >40 mg/dl) (12). Dietary Phe intake was adjusted to achieve blood Phe levels between 2-6 mg/dl in patients <12 years old and between 2-10 mg/dl in patients >12 years old (13). This level of control was considered as appropriate, and blood Phe less than 2 mg/dl was considered as overtreatment (13). Plasma Phe concentrations were measured by the fluorometric method, which is a quantitative and precise method (14). The mean of plasma Phe concentration for each patient was calculated, considering the maximum Phe measurements throughout a year.

Informed consent was obtained orally from the parents and patients who were mature and not mentally retarded.

We also compared anthropometric measures with those of the control group separately for patients who were diagnosed in screening and patients who were diagnosed after presentation of clinical signs and symptoms, we also compared anthropometric indexes in patients who were equal or less than 2 years old and patients who were more than 2 years old with the age-matched control group separately.

**Statistical method:**

This cross-sectional study was performed from 2015 to 2017 in PKU clinic affiliated to Mofid Children’s Hospital

Data were analyzed in SPSS version 22. Distribution of values assessed by One Sample Kolmogorov-Smirnov test and distribution of all values were abnormal, except for midparental height z-score. Mann-Whitney test was used for analyzing the data. *P*-value (predictive value) <0.05 was considered statistically significant. We used correlation analysis to assess the effect of pretreatment and intratreatment phenylalanine levels on anthropometric indexes.

## Results

 Overall, 209 PKU patients (103 males, 106 females; mean age 9.29 ± 8.7 years, range 1 month to 44 years) were enrolled in our study. In general, 130 patients were diagnosed by newborn screening and 79 patients were diagnosed when they became symptomatic before the screening program. Our control group consisted of 216 age and sex-matched controls (mean age: 8.98 ± 8.62 years, range 1 month to 44 years, median years) selected from normal children and adults. Phenylketonuria patients consisted of patients who were diagnosed before screening based on clinical manifestations (79 patients, mean age: 18.3 ± 7.74 years, age range: 4.16 to 44 years) and patients who were diagnosed by the newborn screening program before presenting clinical manifestations (128 patients, mean age: 3.75± 2.21 years, age range: 0.12 to15 years). 

Anthropometric measures of patients were compared with those of the control group in this study.

Mean, median, and standard deviation of weight z-score, height z-score, HC z-score, and BMI z-score of PKU patients and controls are summarized in Table 1.

Phenylketonuria patients versus control group

Weight SDS, height SDS, BMI SDS, weight for height SDS and HC SDS of all the PKU patients were compared with the control group.

There were significant differences between the PKU patients and the control group in HC SDS (z-score) and weight for height SDS (P=0.000 for both variables). Mean HC in the PKU patients was - 0.68± 1.34 cm, whereas this value was 0.081± 0.94 cm in the control group. Mean weight for height z-score in the PKU patients was 1.21± 2.05, whereas this value was 0.51± 1.76 in the control group (Table1). We did not find any significant differences in weight SDS, BMI SDS, and height SDS between the cases and controls. 

Difference between patients’ height SDS and mid-parental height SDS was not significantly different in patients (mean ± SD: 1.9±1.14) and the control group (mean ± SD: 2±1.05) (P=0.81). 

**Phenylketonuria** **patients 2 years old and lower versus age and sex-matched controls**

Weight SDS, height SDS, weight for height SDS, and HC SDS were compared between PKU patients who were less than 2 years old and age and sex matched controls. We did not find any statistically significant difference in any of the anthropometric indexes between cases and controls. Descriptive statistics are summarized in Table 2.

**Phenylketonuria** **patients with more than 2 years old versus control group**

Weight SDS, height SDS, BMI SDS, HC SDS and difference between height SDS and mid-parental height SDS were compared between cases and the age and sex-matched control group.

Head circumference SDS and weight for height SDS were significantly different between the two groups. *P*-value was 0.000 for both variables. The mean HC z-scores in case and control groups were -0.75 and -0.1, respectively. Weight for height SDS was also significantly different between patients and controls (P=0.000). Mean weight for height SDS was -0.89±2.1 and -0.15±1.7 in patients and controls, respectively. Thus, PKU patients had a higher weight for height SDS in comparison with the control group. Other anthropometric indexes were not significantly different in patients and controls. Descriptive statistics are summarized in Table 2.

**Phenylketonuria** **patients who were diagnosed in newborn screening versus patients who were diagnosed after presenting clinical signs and symptoms**

We analyzed patients who were diagnosed in newborn screening and those diagnosed after presenting clinical manifestations before newborn screening program separately with regard to probable differences in anthropometric indexes.

Body mass index SDS, weight SDS, weight for height SDS and HC SDS were significantly different in patients who were diagnosed during newborn screening in comparison with the age and sex-matched control group. Mean HC was significantly lower in PKU patients, while BMI SDS, weight SDS, and weight for height SDS were significantly higher in PKU patients in comparison with the control group. Height SDS and difference between patients’ height SDS and mid parental height SDS were not significantly different. P-values are presented in Table 3.

 Weight SDS, BMI SDS, height SDS, difference between patients’ height SDS and mid parental height SDS and HC SDS were significantly different in patients who were diagnosed after presenting clinical manifestations in comparison with the control group. The means of HC SDS, BMI, height SDS and difference between patients’ height SDS and mid parental height SDS were significantly lower in comparison with the control group, while mean weight SDS was significant higher compared to that of the controls. Values are demonstrated in Table 3. 

Weight SDS, BMI SDS, height SDS, difference between patients’ height SDS and mid parental height SDS and HC SDS were significantly higher in patients who were diagnosed in the screening program in comparison with patients who were diagnosed after presenting clinical manifestations (P=0.001, 0.033, 0.01, 0.000, respectively). Descriptive statistics are summarized in Table 3.

**Pretreatment and mean plasma phenylalanine concentrations:**

The mean of patient’s pretreatment phenylalanine (phenylalanine of the time of diagnosis) was 21.24 ± 8.86 mg/dl (range: 7 to 47).

There was a significant negative correlation between HC z-score and pretreatment Phe concentrations (r= -0.445, P=0.01).

We did not find any correlation between pretreatment Phe and other anthropometric measures.

There was a significant positive correlation between mean phenylalanine and weight SDS (r=0.24, P=0.001) and BMI SDS (r=0.22, P=0.006) Mean intratreatment phenylalanine was not significantly correlated with height SDS, HC SDS, and weight for height SDS. 

## Discussion

Phenylalanine-restricted diet is the most important treatment in classical PKU until now. Several studies have been designed to evaluate the effect of diet therapy on PKU patients’ growth. Interestingly, different results have been reported. Therefore, we designed this case-control study to compare growth in PKU patients who were under diet therapy with normal age and sex-matched Iranian children. 

In this study, we found that Iranian PKU patients were not totally different in weight and height parameters, but were retarded in HC, and weight for height z-score was higher in PKU patients.

 We found no difference between mean weight z-scores, height z-score, and BMI z-score in PKU patients and controls, but when we analyzed patients who were diagnosed during screening and before screening separately, we found that mean HC was significantly lower in PKU patients, while BMI SDS, weight SDS, and weight for height SDS were significantly higher in patients diagnosed in newborn screening program in comparison with the controls. Height SDS and difference between patients’ height SDS and mid parental height SDS were no significant different in this group of patients in comparison with the control group. It shows that HC is affected in PKU patients in spite of Phe restriction diet because of inevitable variation in Phe level even by dieting. It was more prominent in patients who were more than 2 years old probably because of more difficulty to force children in this age group to have commitment to a specific diet. 

Higher mean levels in weight SDS, weight for height SDS, and BMI SDS in this group of patients probably result in high carbohydrate diet in contest for providing appropriate calorie and change in patients’ appetite. Surprisingly, height SDS was not affected in this group of patients in spite of Phe (protein) restricted diet. In patients who were diagnosed after presenting clinical manifestations before newborn screening program, HC SDS, BMI SDS, height SDS and difference between patients’ height SDS and mid parental height SDS had significant lower means in comparison with the control group, while weight SDS was significantly higher relative to the controls. It may be due to neurologic defects in patients that could affect patients’ nutrition and physical activity as well as parental cares. Although there is dietary phenylalanine restriction (low protein diet) in these patients which usually causes height reduction, we tried to reach an adequate amount of calorie by consuming more special formula or foods (high carbohydrate diet) that have less phenylalanine, resulting in higher weight SDS. In this group, BMI SDS was low because height was affected.

Huemer et al. and Holm et al. found no significant difference in weight of PKU patients and the normal population (7, 15). This result was also true about weight, length, and HC of 35 infants studied by Acosta et al. However, plasma phenylalanine concentration was not correlated with growth (16). 

On the contrary, McBurnie et al. found that mean weight was significantly different (p<0.05) between PKU and NCHS groups at most ages for both sexes (17). Also, Dobbelare et al. found PKU patients were shorter and lighter than the French reference population (18). In a recent study, Luis Aldamiz-Echevarria et al. reported early-stage weight and height growth retardation in PKU patients in comparison with the Spanish reference population (4). Administration of tetrahydrobiopterin did not correct growth (4, 19). Egyptian studies reported normal weight and height growth in PKU patients and reported that many patients became obese or overweight (20, 21). Susceptibility to increased fat mass was reported in PKU patients, especially in female patients above 11 years old in the Netherlands (8), but Rocha et al. did not find any statistically significant difference in fat mass and incidence of overweight and obesity and metabolic syndrome between patients and controls in another study (22). 

Dobbelaer et al. reported that PKU patients were moderately but significantly shorter (H/A z-score varied from -2.12 to 1.61; mean: -0.49) and lighter (W/A z-score varied from -2.58 to 1.49; mean: -0.71) than the French reference population. Body composition in patients was not different from that of controls matched for age and sex (6). Rocha et al. reported normal growth in 89 PKU patients in comparison with controls (7, 9).

Inconsistent results in these studies may be due to different techniques of diet therapy in aspects of restriction of Phe and protein and usage of carbohydrates and fat and calculation of calories. Also, rigidity in cradling phenylalanine in advised levels and patients’ adherence to therapy may be effective in different growth patterns in PKU patients. 

We found no significant difference between height z-scores of patients and controls less than 2 years old, and the cause may be achieving sufficient calories and supplements by patients. Also, dietary adherence is much easier in patients less than 2 years old. There was no significant difference in height z-score between patients above 2 years old and age and sex-matched controls. It shows that chronic low protein intake and micronutrient deficiency could not affect linear growth in Iranian PKU patients. 

**Table 1:** Weight, height and head circumference z-scores of the phenylketonuria patients and the control group

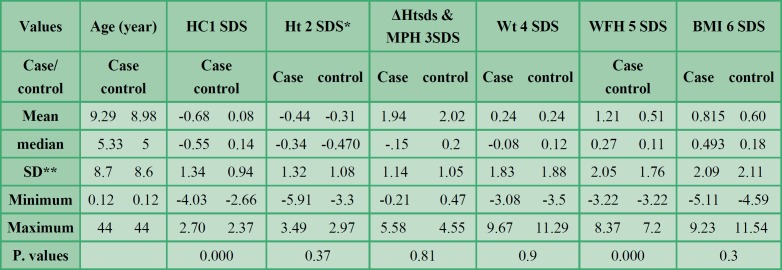

^1 ^HC: head circumference

^2 ^Ht: Height

^3^ Δ Htsds & MPH: difference between height SDS and mid parental height SDS

^4 ^Wt: weight

^5^ WFH: weight for height

^6 ^BMI: body mass index

*SDS: standard deviation score: z-score

** SD: standard deviation

**Table 2:** Weight, height, head circumference, weight for height and body mass index z-scores of phenylketonuria patients and the control group with ages <= 2 years and > 2 years

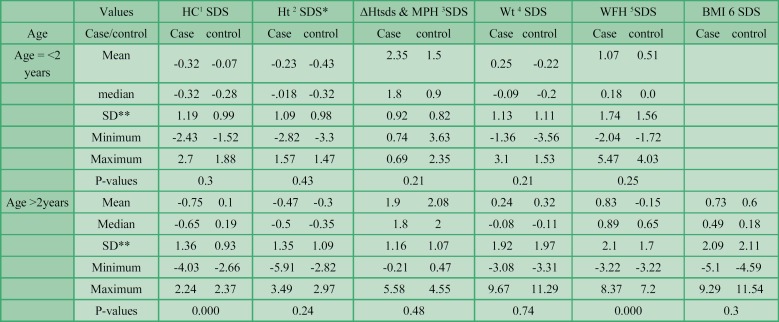

**Table 3:** Weight, height, head circumference, weight for height and BMI z-scores of phenylketonuria patients who were diagnosed in newborn screening and patients who were diagnosed after presenting clinical manifestations

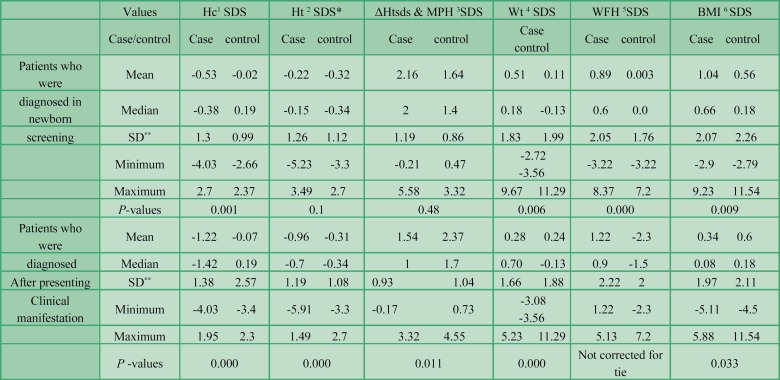

^1 ^Hc: head circumference

^2 ^Ht: height

^3^ Δ Htsds & MPH: difference between height SDS and mid parental height SDS

^4 ^Wt: weight

^5^ WFH: weight for height

^6 ^BMI: body mass index

*SDS: standard deviation score: z-score

** SD: standard deviation

Hoeksma showed that there was no relationship between energy and protein intake and height growth (10). Furthermore, lower height standard deviation score may be due to growth hormone resistance that is found in almost all chronic diseases. However, IGF1, IGFBP3, and thyroid hormone levels were within the normal range and no relationship was found between plasma Phe concentrations, protein or caloric intake and the presence of growth retardation in a study by Dobbelaere et al. (18). Comparison of height with family data by Holm et al. showed that children with PKU grew as expected for their genetic endowment as we showed in our study in patients who were diagnosed in newborn screening (23). 

In a longitudinal study, the mean height SDS (z-score) was not significantly different at baseline, but it decreased during the second year of life and weight for height SDS was in normal range in these patients (24). Another study revealed no significant difference in height z-score in patients less than 19 years old, while height z-score was significantly lower in patients above 19 years old (9). Aldamiz-Echevarria et al. reported different growth manners in different ages in PKU patients. There was a positive correlation between height growth and height standard deviation score in PKU patients in their study (4).

In our study, there was a significant decrease in patients’ HC than in controls', however, by comparing patients and controls in the two groups, the difference was significant in patients above 2 years old. McBurnie et al. found that HC in PKU children tended to be lower than NCHS standards; however, the maximum difference at any age was less than 0.5 cm (17). Verkerk et al. found PKU patients had smaller HCs than the normal population, and more growth retardation is observed in their first three years of life (25).

Head circumference was not smaller in patients less than 2 years old, this might result from tighter control and screening in lower ages. The negative significant correlation between HC and pretreatment plasma Phe was in contrast with findings of other studies (17). Hoeksma et al. found a positive correlation between natural protein intake and HC growth in a study of 174 0 to 3-year-old patients (26). While finding no correlation between growth parameters and mean plasma Phe levels (a sign of dietary adherence) could reveal that disease severity (pretreatment Phe levels) is a more effective factor in HC growth.

There was no difference between weight for height in PKU patients and controls (less than 2 y/o), and this means that patients are totally in the normal range or weight and height were appropriately decreased (26). According to our study, patients’ weight SDS, BMI SDS, and weight for height SDS were higher in comparison with the control group probably resulting in high carbohydrate levels. Even it was reported by Verkerk et al. that there were fewer children lower and higher than 10th and 90th weight for height percentile than expected (25).

However, no statistically significant difference between PKU patients and controls was reported by Huemer (7). Dobbelaere et al. also found body composition was not different from that of controls matched for age and sex (18). 

Another interesting result in our study was absence of correlation between mean phenylalanine level during treatment and growth indexes; however, pretreatment phenylalanine level had a negative correlation HC SDS in patients.

**In Conclusion** **, **The most important anthropometric index that was affected in PKU patients was HC, especially in patients with more than 2 years of age in spite of the fact that patients were under Phe restriction diet. Significantly higher weight z-score, BMI z-score, and weight for height z-score were seen in patients who were under early onset nutritional treatment probably because of low protein and high carbohydrate diet. Height z-score and BMI z-score were significantly lower in patients diagnosed after clinical presentation probably because of the existing sequels affecting nutrition and activity of patients, while weight SDS was significantly higher. Differences in anthropometric index changes that observed in different studies may be due to disparate diet protocols, availability of various specific products and micronutrient substitution.
